# *In vivo* photoacoustic guidance of stem cell injection and delivery for regenerative spinal cord therapies

**DOI:** 10.1117/1.NPh.7.3.030501

**Published:** 2020-07-29

**Authors:** Kelsey P. Kubelick, Stanislav Y. Emelianov

**Affiliations:** a Georgia Institute of Technology, Emory University School of Medicine, Wallace H. Coulter Department of Biomedical Engineering, Atlanta, Georgia, United States; b Georgia Institute of Technology, School of Electrical and Computer Engineering, Atlanta, Georgia, United States

**Keywords:** ultrasound, photoacoustic imaging, magnetic resonance imaging, stem cell therapy, spinal cord, nanoparticles

## Abstract

**Significance:** Stem cell therapies are of interest for treating a variety of neurodegenerative diseases and injuries of the spinal cord. However, the lack of techniques for longitudinal monitoring of stem cell therapy progression is inhibiting clinical translation.

**Aim:** The goal of this study is to demonstrate an intraoperative imaging approach to guide stem cell injection to the spinal cord *in vivo*. Results may ultimately support the development of an imaging tool that spans intra- or postoperative environments to guide therapy throughout treatment.

**Approach:** Stem cells were labeled with Prussian blue nanocubes (PBNCs) to facilitate combined ultrasound and photoacoustic (US/PA) imaging to visualize stem cell injection and delivery to the spinal cord *in vivo*. US/PA results were confirmed by magnetic resonance imaging (MRI) and histology.

**Results:** Real-time intraoperative US/PA image-guided injection of PBNC-labeled stem cells and three-dimensional volumetric images of injection provided feedback necessary for successful delivery of therapeutics into the spinal cord. Postoperative MRI confirmed delivery of PBNC-labeled stem cells.

**Conclusions:** The nanoparticle-augmented US/PA approach successfully detected injection and delivery of stem cells into the spinal cord, confirmed by MRI. Our work demonstrated *in vivo* feasibility, which is a critical step toward the development of a US/PA/MRI platform to monitor regenerative spinal cord therapies.

## 1 Introduction

In spite of great potential and substantial research, few stem cell therapies have reached clinical implementation. The lack of real-time longitudinal imaging feedback during procedures limits therapy development, making it challenging to evaluate outcomes from a research and clinical standpoint. In particular, stem cell therapies of the spinal cord could benefit from intra- and postoperative image guidance.^1^^–^^5^ In a recent clinical study, amyotrophic lateral sclerosis patients received stem cell treatments through direct injection into the spinal cord, which is appealing due to better outcomes in spite of high risk associated with the invasive surgical procedure.^2^^,^^5^^–^^8^ Real-time intraoperative image guidance of needle insertion is desired to improve procedure safety and guide stem cell delivery to the tissue target to validate success prior to completing the surgery.^1^^,^^3^^,^^4^ Next, postoperative monitoring is valuable to track stem cell location and function over time to evaluate therapy progression.

Researchers have investigated the use of magnetic resonance imaging (MRI) augmented with superparamagnetic iron oxide nanoparticles (SPIONs) to label stem cells for longitudinal, postoperative monitoring.^9^ However, high cost, large footprint, operating room incompatibility, and slow image acquisition limit MRI use in an intraoperative setting, where imaging tools are critically needed to guide stem cell therapies of the spinal cord. To address the need for intraoperative guidance, we initially proposed an ultrasound (US) and photoacoustic (PA) imaging approach to detect gold nanosphere (AuNS)-labeled stem cells in the spinal cord.^3^ Although feasibility was validated in excised rodent spinal cords *ex vivo*, the AuNS-based approach does not allow for postoperative MRI guidance. Therefore, we developed an alternative imaging approach to detect stem cells in the spinal cord using a unique nanoconstruct, Prussian blue nanocubes (PBNCs).^4^

PBNCs are composed of Prussian blue dye, which is clinically approved to treat heavy metal poisoning, and iron oxide nanoparticles, which are under assessment in clinical trials or already approved for certain imaging applications.^10^^,^^11^ The material composition of PBNCs results in PA and MR imaging contrast.^10^^,^^12^ Thus, US/PA imaging can be used for intraoperative guidance of PBNC-labeled stem cell injection and delivery followed by MRI for postoperative verification and longitudinal monitoring.^4^^,^^13^ The high resolution and contrast, fast image acquisition, small footprint, portability, relatively low cost, and operating room compatibility make US/PA imaging ideal for intraoperative use.^14^^,^^15^ Furthermore, US/PA imaging tools have been successfully demonstrated in several different applications of stem cell detection^12^^,^^16^^–^^19^ or procedure guidance outside of the spinal cord, including sentinel lymph node biopsy,^20^^,^^21^ robotic surgeries,^22^ and tumor resection.^20^ Although there is excitement surrounding US/PA image-guidance of stem cell therapies, greater penetration depth and clinical familiarity of MRI for spinal cord applications warrants its continued use, especially at pre- and postoperative stages. The combination of a US/PA/MRI tool for stem cell tracking in the spinal cord creates an imaging approach that can be utilized throughout the course of treatment in the spinal cord.

Our prior work evaluated PBNC synthesis, optical and magnetic properties, and multimodal contrast of PBNC-labeled stem cells *in vitro*.^10^ Our earlier *ex vivo* studies in excised spinal cords validated PA/MR agreement.^4^ However, *in vivo* translation is faced with many challenges by attempting to add US/PA to a complex, risky surgical procedure, which is further magnified by motion, background signals, increased imaging depth, and the need for fast image acquisition, among many other factors.

The present work demonstrated an intraoperative US/PA imaging approach for surgical guidance of stem cell therapy of the spinal cord *in vivo*. Our results showed successful real-time intraoperative US/PA imaging of PBNC-labeled stem cells in the spinal cord, followed by postoperative MRI confirmation. Previous *ex vivo* studies using PA imaging in the spine demonstrate the capabilities of new imaging tools to guide surgery and therapies.^3^^,^^4^^,^^13^^,^^23^^,^^24^ Current *in vivo* findings on intraoperative US/PA guidance are vital to supporting further development of the trimodal US/PA/MR imaging approach to monitor stem cell therapies in the spinal cord. Results may extend to other research and clinical spine-related applications in intra- and postoperative settings.

## 2 Materials and Methods

### 2.1 Synthesis of Prussian Blue Nanocubes

All chemicals were used as received and were purchased from Sigma-Aldrich unless noted otherwise. Dextran-coated PBNCs with an edge length of ∼200  nm and a peak absorption of ∼750  nm wavelength (Supplemental Fig. S1) were synthesized in-house using methods described elsewhere.^10^ Briefly, reactant and catalyst solutions were prepared in advance; they consisted of 5% potassium hexacyanoferrate (II) trihydrate by mass in deionized ultrafiltered (DIUF) water and 1.85% HCl in DIUF water, respectively. SPIONs (Ocean Nanotech; 60 mg) were added to 150 mL of DIUF water. Next, 7.5 mL of reactant and 2.5 mL of catalyst were added to the SPION solution and stirred for at least 1 h. PBNCs were characterized using transmission electron microscopy (TEM; Hitachi HT7700) and UV-Vis spectrophotometry (Synergy HT microplate reader, Bio-Tek Instruments).

### 2.2 Stem Cell Labeling Protocol

Human adipose-derived mesenchymal stem cells (MSCs; Lonza) were labeled with PBNCs using previously reported methods,^4^ and minimal impact on stem cell viability and multipotency following labeling was observed through *in vitro* assessments, which are commonly performed to independently assess nanoparticle impact on cellular function and anticipate effects *in vivo*.^25^ Briefly, at roughly 80% confluence, MSCs were incubated with PBNC-containing media overnight. PBNCs were dispersed in the media at an optical density of 2, corresponding to roughly 53  μg Fe/mL of media. The following day, PBNC-labeled MSCs were washed at least 3 times with phosphate buffered saline (PBS) to remove excess particles. Next, the PBNC-labeled MSCs were detached from the tissue culture flask using Trypsin-EDTA followed by centrifugation (400×g, 5 min) to further eliminate extracellular PBNCs by size separation. PBNC-labeled MSCs were suspended in PBS at 10  k cells/μL, a clinically relevant concentration and stored on ice prior to injection.

For histological analysis, MSCs were also incubated with 10  μM fluorescent dye (CellTracker Green CMFDA Dye, Thermo Fisher Scientific) for 45 min. Thus MSCs were double-labeled with PBNCs and a fluorescent dye in culture prior to injection into the spinal cord. Cell uptake of PBNCs was confirmed using brightfield microscopy (Axio Observer, Zeiss) *in vitro*.

### 2.3 Imaging Systems

Combined US/PA images were acquired at 5  frames/s using a Vevo LAZR (FUJIFILM VisualSonics, Inc.) imaging system and a 20-MHz linear array transducer coupled with an optical fiber bundle (LZ250). The laser source was a Q-switched Nd:YAG pumped optical parametric oscillator laser (pulse repetition frequency=20  Hz, 7 ns pulse duration, and 680 to 970 nm wavelength). Three-dimensional (3-D) US/PA images were compiled from two-dimensional (2-D) cross-sectional US/PA views acquired every 120  μm using a motorized translational stage.

MR images were acquired using a 7T preclinical system (Bruker PharmaScan) and a gradient coil with an inner diameter of 60 mm. The built-in T2-Turbo RARE pulse sequence was used to produce images with T2-weighted contrast. The repetition (TR) and echo times (TE) were approximately TR=4250  ms and TE=33  ms.

### 2.4 Surgical Procedure

All studies involving animals were conducted following guidelines of the Institutional Animal Care and Use Committee (IACUC) of the Georgia Institute of Technology.

To demonstrate successful implementation of the imaging tool, the study was conducted in 6 female rats (four 6- to 8-weeks old Sprague Dawley rats and two 43- to 45-weeks old nude rats). One animal died from surgical complications prior to completion of the imaging study. Rats were anesthetized and prepared for surgery by removing hair and sterilizing the exposed skin. Prior to incision, sustained release buprenorphine (0.6 to 0.8  mg/kg) was administered for postoperative analgesia.

A lumbar laminectomy was performed to expose the spinal cord and allow direct injection of PBNC-labeled MSCs into the tissue according to previous methods.^1^^,^^3^^,^^8^ Up to 5  μL of PBNC-labeled MSCs suspended at 10k  cells/μL were directly injected into the spinal cord *in vivo* at a rate of 16  nL/s using a 33G syringe attached to an ultramicropump (World Precision Instruments). The needle remained in the spinal cord for 5-min postinjection to prevent reflux. After the stem cell injection, the muscle was sutured back over the spinal cord, followed by the skin. The bone was not replaced, per the clinical protocol. The surgical and intraoperative imaging setup is shown in Supplemental Fig. S2.

### 2.5 *In Vivo* Intraoperative Imaging with US/PA

During needle insertion and injection of PBNC-labeled stem cells, US/PA datasets were acquired at 750 nm wavelength in real time. After the stem cell injection, the needle was removed, and 3-D US/PA datasets at 750 nm wavelength were acquired prior to closing the incision. Following beamforming and envelope detection using the built-in Vevo LAZR imaging system protocols, combined US/PA datasets were exported to MATLAB (Mathworks Inc.) for additional postprocessing using the methods developed in our previous *ex vivo* studies.^4^ During needle insertion, PA data was segmented to primarily color the needle shaft red. During the cell injection, PA data were further segmented to distinguish PBNC-labeled MSCs, which were primarily colored blue. Three-dimensional US/PA datasets were analyzed using AMIRA (Thermo Fisher Scientific) to display volumetric images.

### 2.6 *In Vivo* Postoperative Imaging with MRI

T2-weighted MR images of PBNC-labeled MSCs were acquired *in vivo* 24 h after intraoperative US/PA imaging, as required by IACUC guidelines. Rats were anesthetized and secured in a cylindrical holder that was inserted into the imaging bore of the 7T MR scanner. Axial cross-sectional images with a slice width of 1 mm were acquired along the length of the spinal cord *in vivo*. MR images were visualized using the built-in Bruker PhamaScan software, and the image display was adjusted to maximize contrast between the PBNC-labeled stem cells and background tissue.

Following *in vivo* MRI, rats were sacrificed, and the spinal cord was excised for histology. Tissue sections were stained with eosin (VWR International) for brightfield microscopy (Axio Observer, Zeiss) or DAPI [2-(4-Amidinophenyl)-6-indolecarbamidine dihydrochloride; Thermo Fisher Scientific] for fluorescent confocal microscopy (LSM 700, Zeiss).

## 3 Results

**Fig. 1:**
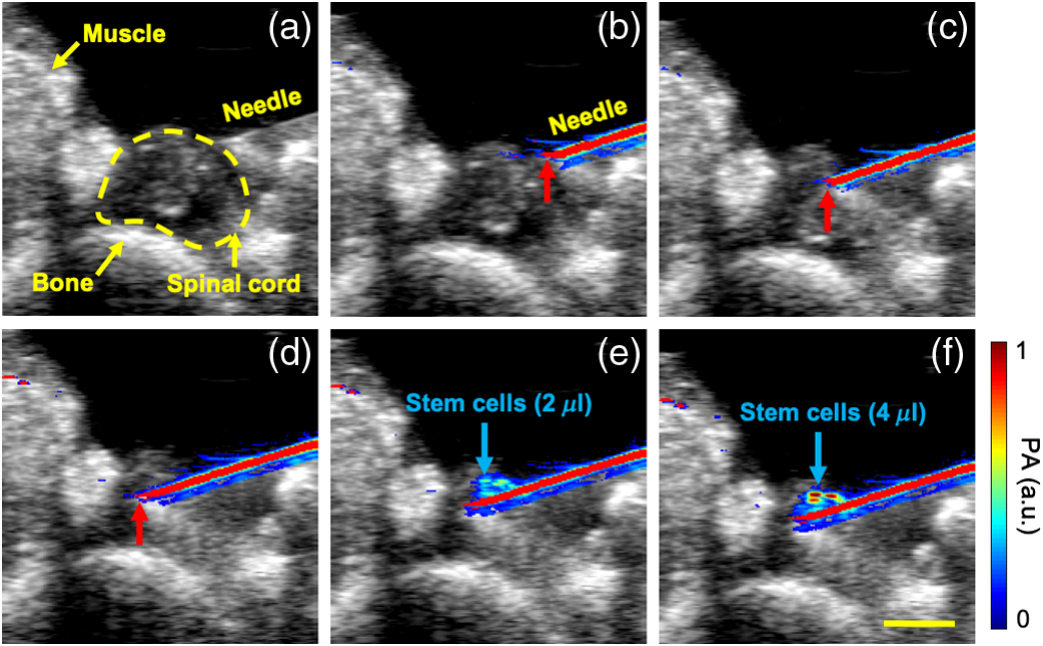
*In vivo* intraoperative US and PA image-guided injection of PBNC-labeled stem cells. US (20 MHz, grayscale) and PA (750 nm wavelength, color scale) images were acquired in real time. US images alone provide anatomical context of imaged volume including spinal cord, muscle, and bone (a). High-contrast PA signals identified the needle shaft (primarily colored red) during insertion into the spinal cord (b)–(d). Red arrows indicate the needle tip. Following needle insertion, PBNC-labeled stem cells (primarily colored blue) were injected into the spinal cord [(d)–(f): 0, 2, and 4  μL, respectively]. Scale bar=2  mm.

Real-time intraoperative US/PA images were acquired as the needle was brought into the field of view and inserted into the spinal cord *in vivo* (Fig. 1). The high-contrast PA images depicted the needle shaft and location of the needle tip [Figs. 1(b)–1(d)], which assisted in visualizing needle placement *in vivo*. After inserting the needle and positioning the tip at a desired location within the spinal cord, US/PA images were continuously acquired in real time during the injection of PBNC-labeled MSCs. Accumulation of PBNC-labeled MSCs was visible surrounding the needle tip based on the increase in PA signal with increasing injection volume *in vivo* [Figs. 1(e) and 1(f)].

**Fig. 2:**
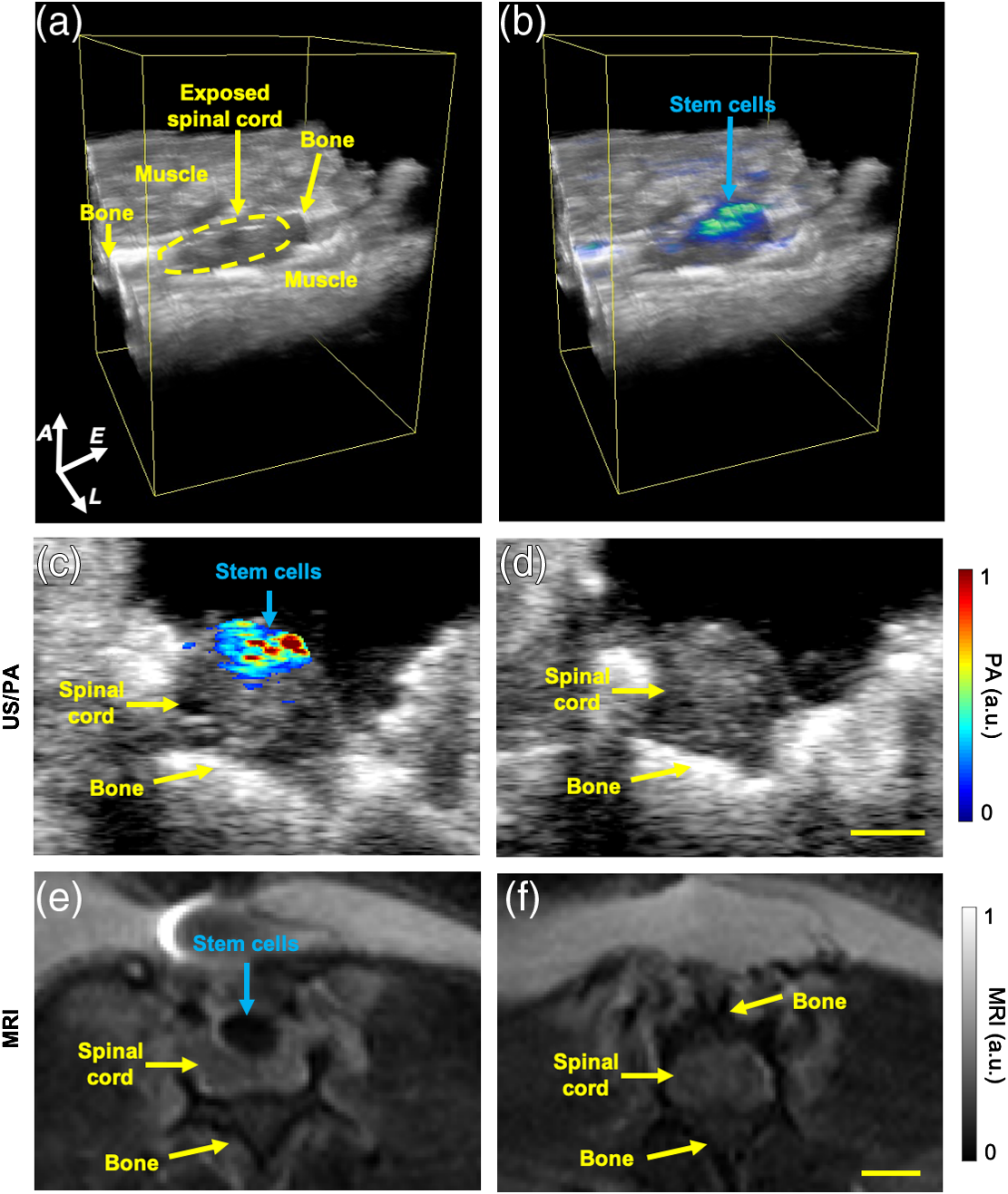
*In vivo* US, PA, and MRI of PBNC-labeled stem cells. After cell injection and needle removal, (a) volumetric US and (b) US/PA images visualized the bolus of injected PBNC-labeled stem cells and their retention in the spinal cord. The elevational scan distance was ∼13  mm (A, axial; L, lateral; and E, elevational). Axial cross-sectional views of PBNC-labeled MSCs obtained from (c) intraoperative US/PA images and (e) postoperative MR images acquired 24 h later were compared qualitatively to confirm spatial correlation of the injected bolus of stem cells. Baseline (d) US/PA and (f) MRI of regions without stem cells. Baseline MRI was at a region where a laminectomy was not performed, indicated by the presence of overlying bone (f). Scale bar=2  mm.

After needle removal, additional intraoperative data were acquired prior to closing the incision. Three-dimensional US/PA volumetric images visualized the stem cell injection bolus [Figs. 2(a) and 2(b)]. Within 10 min-postinjection, spreading of the stem cell injection along the spinal cord away from the needle insertion point was noted [Fig. 2(b)]. In addition, reflux of stem cells was likely based on superficial localization in the spinal cord tissue, observed in 3-D [Fig. 2(b)] and 2-D [Fig. 2(c)] images. Two-dimensional intraoperative US/PA images were then compared with postoperative *in vivo* MR images acquired 24 h later. The bolus of PBNC-labeled MSCs was visible at similar locations in intraoperative US/PA [Fig. 2(c)] and postoperative MR [Fig. 2(e)] images, which were spatially correlated at a qualitative level. PA and MR images from each animal (n=5) were further analyzed to compare detection of the stem cell injection bolus (Supplemental Fig. S3). Our results validated *in vivo* US/PA surgical guidance and agreement with postoperative MRI, indicating potential for monitoring throughout the course of treatment.

**Fig. 3:**
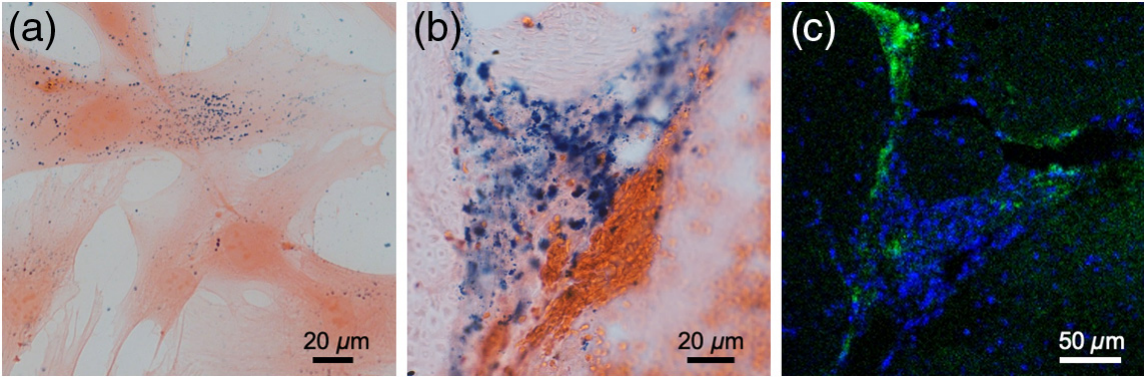
Histological analysis of PBNC-labeled stem cells. Stem cells were double-labeled with PBNCs and a green fluorescent dye prior to injection into the spinal cord. Brightfield microscopy of (a) PBNC-labeled stem cells and (b) eosin-stained spinal cord tissue. Blue pigment indicates PBNCs. (c) Confocal microscopy of DAPI stained spinal cord tissue and double-labeled stem cells.

Following *in vivo* studies, histological analysis further validated our imaging results (Fig. 3). *In vitro* studies verified successful labeling of MSCs with PBNCs, indicated by co-localization of pink cytoplasm with blue pigmented PBNCs [Fig. 3(a)]. Brightfield microscopy of excised spinal cords similarly showed blue PBNC-labeled MSCs [Fig. 3(b)]. Confocal fluorescent microscopy further confirmed the presence of PBNC-labeled MSCs, which were double-labeled with green fluorescent dye.

## 4 Discussion

Results showed *in vivo* feasibility of a nanoparticle-augmented imaging approach for surgical guidance, specifically needle insertion and delivery, of stem cell therapies in the spinal cord. Prior to surgery, stem cells were labeled with PBNCs, a contrast agent for PA and MRI.^10^ The multimodal contrast creates a unique opportunity to develop a US, PA, and MRI approach that is better suited to monitoring stem cells in the spinal cord intra- and postoperatively. This work expands on prior *ex vivo* results by demonstrating successful *in vivo* translation.^3^^,^^4^

Combined US/PA imaging was implemented intraoperatively to guide needle insertion and injection of PBNC-labeled stem cells into the spinal cord *in vivo*.^26^ Three-dimensional US/PA volumetric images of the injection bolus gave necessary spatial context to assess stem cell location and retention at the injection site, which may provide feedback to improve outcomes by informing real time modifications prior to completing surgery.

Next, *in vivo* MR images were acquired 24 h later to validate US/PA results and confirm detection of PBNC-labeled stem cells in a postoperative environment. As the gold standard for spinal cord imaging, MR images of PBNC-labeled MSCs were critical for validating US/PA results. However, beyond serving as a control, the multimodal contrast of PBNCs provides an excellent opportunity for trimodal US/PA/MR imaging of stem cells in the spinal cord to extend imaging capabilities throughout the course of treatment by utilizing intraoperative US/PA followed by postoperative MRI.

There are several opportunities for future development. For guiding needle insertion intraoperatively, modifications should be made to alter the orientation of the needle shaft and transducer. In the current setup, angled needle insertion [Fig. 1(b) and Supplemental Fig. S2] was required to accommodate the injection syringe and US/PA imaging transducer over the small region of exposed spinal cord in a rodent. To more easily reach a specific tissue target, the needle should be inserted straight into the spinal cord at a 90-deg angle relative to the coronal plane. Others have developed a spinal cord derrick to stabilize the injection syringe during needle insertion into the spinal cord.^27^ A similar scaffold may be used to maintain orientation and to stabilize the US/PA imaging transducer and injection needle on the same fixture, which can improve US/PA guidance by facilitating rapid, consistent imaging in a time-sensitive, surgical setting.

Quantification of stem cell delivery, such as concentration and dose delivered, could also extend intraoperative utility.^3^^,^^4^ In this study, single-wavelength PA datasets were acquired to minimize image acquisition time. Quantification using PA signal amplitude is not ideal due to variability resulting from transducer position, animal motion, or laser fluence. Multiwavelength PA imaging and spectroscopic analysis are needed to accurately quantify stem cell delivery, but these strategies require longer image acquisition times.

Postoperative longitudinal monitoring is desired to understand stem cell behavior and therapy progression over time.^9^ Beyond location, longitudinal feedback on the functional status of stem cells can provide valuable information on the regenerative mechanism of action by detecting stem cell viability, differentiation, and proliferation.^28^^–^^30^ Nanosensor-augmented US/PA imaging is inherently well-suited to assess and monitor some cellular functions.^14^^,^^15^ Although many research opportunities remain to develop a clinical US/PA/MRI approach augmented with PBNCs to guide stem cell therapies of the spinal cord, results of the current *in vivo* study were a critical step toward supporting future research.

## 5 Conclusions

Stem cell therapies of the spinal cord could benefit from intra- and postoperative image guidance to provide feedback on the surgical procedure and therapy progression. We developed a nanoparticle-based trimodal imaging approach for image-guided delivery of stem cells in the spinal cord using intraoperative US/PA imaging and postoperative MRI *in vivo*. Stem cells were labeled with PBNCs to produce PA and MRI contrast. Intraoperative US/PA imaging guided needle insertion and injection of PBNC-labeled stem cells into the spinal cord *in vivo*, and 3-D volumetric images visualized stem cell retention at the injection site. After 24 h, *in vivo* MR images validated US/PA results and confirmed detectability of PBNC-labeled stem cells in a postoperative environment. Results represent an important milestone to motivate continued development of imaging approaches for spinal cord therapies and beyond.
