# Supplementary figures and images for: In vivo photoacoustic guidance of stem cell injection and delivery for regenerative spinal cord therapies
Source: Neurophotonics. 2020 Jul 29;7(3):030501. doi: 10.1117/1.NPh.7.3.030501 (PMC7388074; doi:10.1117/1.NPh.7.3.030501)

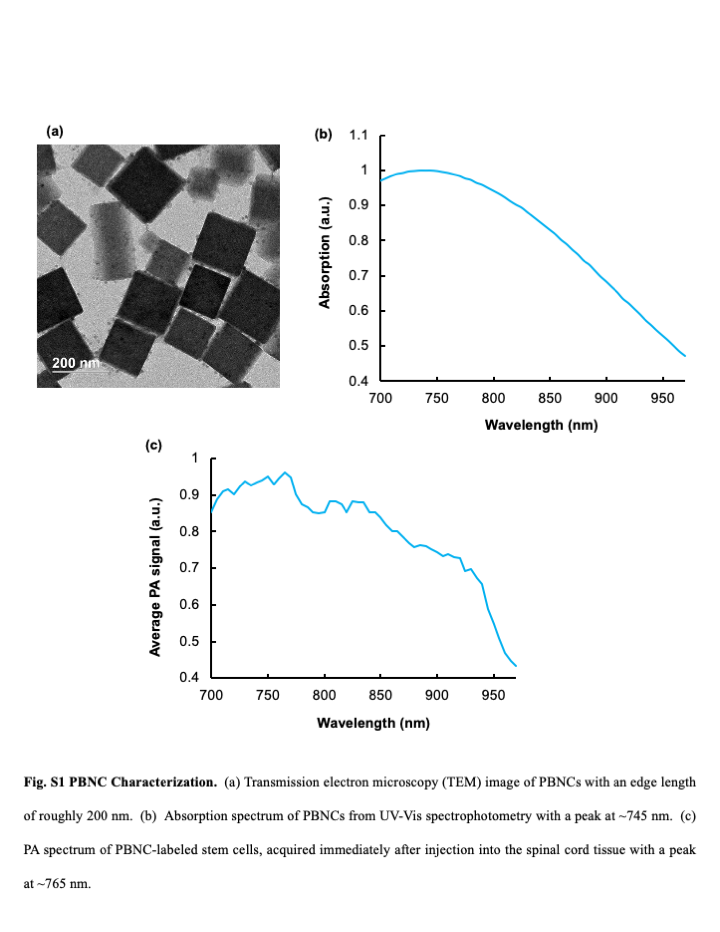

Supplement: Supplementary file 1 [file NPh_007_030501_SD001.tif]

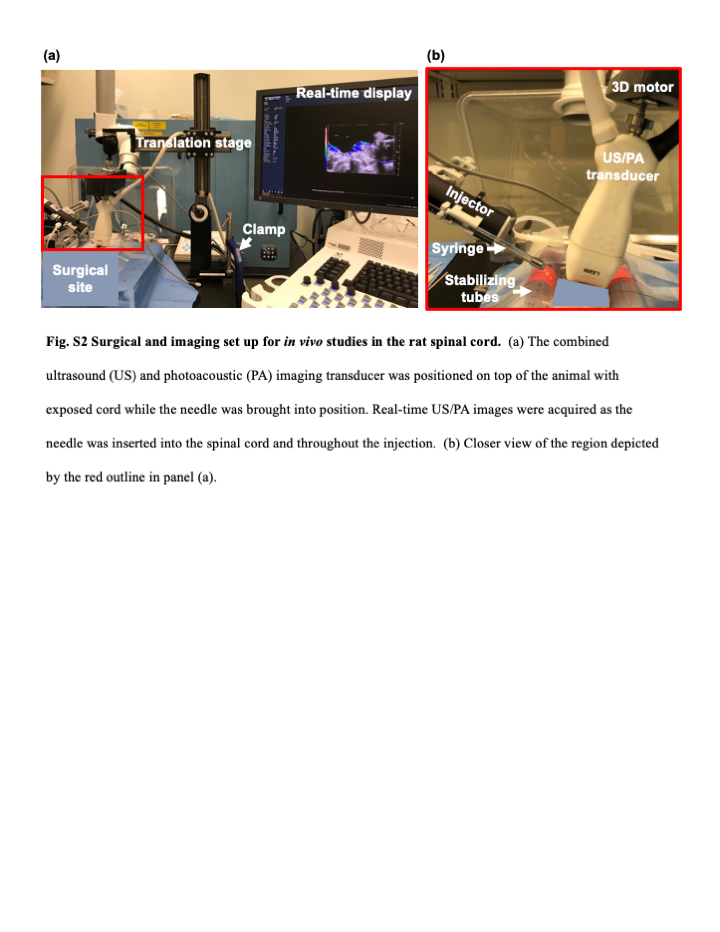

Supplement: Supplementary file 2 [file NPh_007_030501_SD002.tif]
