# Supplementary material for: In vivo photoacoustic guidance of stem cell injection and delivery for regenerative spinal cord therapies
Source: Neurophotonics. 2020 Jul 29;7(3):030501. doi: 10.1117/1.NPh.7.3.030501 (PMC7388074; doi:10.1117/1.NPh.7.3.030501)
Supplement: Supplementary file 3 [file NPh_007_030501_SD003.pdf]

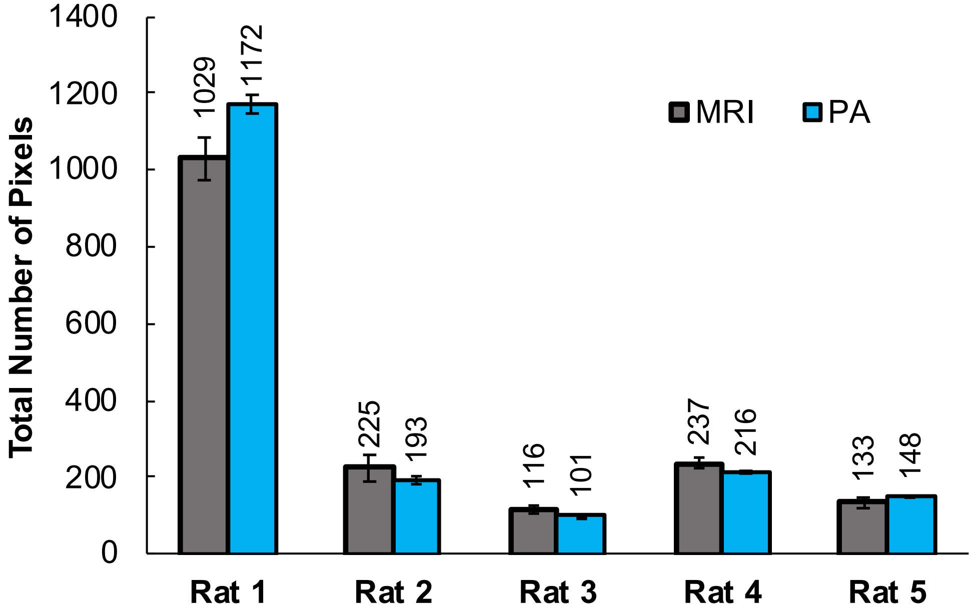

Fig. S3 Photoacoustic (PA) and magnetic resonance imaging (MRI) quantification of the stem cell injection bolus. Intraoperative cross-sectional PA and MR images were processed to calculate the total number of pixels corresponding to the stem cell injection bolus. The area of the spinal cord injection was manually segmented by selecting five independent regions of interest. For each trial, the total number of pixels corresponding to the stem cell injection bolus was calculated. The average was plotted for each animal, and error bars represent +/- one standard deviation. For each animal, intraoperative PA and postoperative MR results were similar, which further supports agreement between modalities and validates the PA/MRI approach. Variability between animals was observed, highlighting the need for intraoperative guidance. Although all animals were subject to identical procedures, uncontrollable factors during surgery, i.e. needle clogging, stem cell reflux, or injection spread along the spinal cord, can impact distribution of the stem cell injection bolus in tissue. This was observed in Rat 1, where a larger injection footprint, and hence a greater number of pixels, corresponding to stem cells was found. Real-time intraoperative image guidance can help visualize this surgical variability to improve procedure consistency.
